# Accuracy of operative time prediction in endometriosis surgery: results of a prospective randomized multicenter pilot study

**DOI:** 10.1007/s00404-026-08545-6

**Published:** 2026-08-06

**Authors:** Marta Duvnjak, Andreas Mueller, Simon Binder, Philipp Guttenberg, Mai Thi Hornaus, Judith Helfenritter, Alexander Boosz, Evgeni Baev

**Affiliations:** 1 Department of Obstetrics and Gynecology, Karlsruhe Municipal Hospital, Karlsruhe, Germany; 2 Department of Obstetrics and Gynecology, ViDia Hospital, Karlsruhe, Germany; 3 Department of Obstetrics and Gynecology, Oberschwabenklinik, Ravensburg, Germany; 4 Friedrich Alexander University of Erlangen–Nuremberg, Erlangen, Germany

**Keywords:** Endometriosis, #ENZIAN, Digital rectal examination, Surgery

## Abstract

**Objective:**

To evaluate whether preoperative clinical Enzian scoring and digital rectal examination improve accuracy of operative time prediction and surgical outcomes in women undergoing surgery for endometriosis.

**Design:**

Prospective, randomized, multicenter pilot trial.

**Setting:**

Three urban teaching hospitals certified as endometriosis centers.

**Patients:**

A total of 107 women undergoing surgery for endometriosis.

**Interventions:**

The participants were randomly assigned to three groups: Group 1 underwent clinical Enzian scoring combined with digital rectal examination (DRE); Group 2 underwent clinical Enzian scoring only; and Group 3 underwent digital rectal examination only. All patients received standard transvaginal ultrasound examinations.

**Main outcome measures:**

The primary endpoint was defined as an absolute difference of ≤ 10 min between the estimated and the actual operative time. Secondary end points included conversion to laparotomy, abandonment of the operation, complete resection rate, need for secondary surgery, correspondence between the preoperative Enzian assessment and intraoperative findings, and postoperative pain (visual analog scale) and patient satisfaction.

**Results:**

The primary endpoint was achieved in 9 patients (23.7%) in Group 1, 16 patients (47.1%) in Group 2, and 15 patients (42.9%) in Group 3. Exploratory comparison of the three groups did not demonstrate a statistically significant difference in the proportion of accurate operative time estimations. Secondary endpoints, including conversion rate, abandonment of the operation, complete resection rate, reoperation rate, and patient-reported outcomes, were also comparable across the groups.

**Conclusions:**

The results demonstrate that neither diagnostic strategy provided a measurable advantage in predicting operative time or improvement of surgical outcome parameters.

**Study registration number:**

DRKS00040760.

## What does this study add to clinical work?

**Table:** 

Omitting diagnostic procedures that do not provide additional clinical benefit may simplify preoperative care and reduce patient burden without compromising the quality of care or surgical safety. In this pilot trial, complex preoperative assessment and classification were not associated with a significant improvement in the accuracy of operative time prediction.	

## Introduction

Endometriosis is a chronic inflammatory gynecological disease characterized by endometrial-like tissue located outside the uterus [1]. The condition affects approximately 10% of women, primarily of reproductive age, and leads to a sustained reduction in their quality of life [2, 3]. Endometriosis is usually located within the abdominal cavity and can be classified into three different forms: peritoneal, ovarian, and deeply infiltrating endometriosis [4]. Accurate preoperative diagnosis of endometriosis continues to be challenging, due to the heterogeneous presentation of the disease and the variable depth and localization of the lesions [4, 5]. Traditionally, laparoscopy with histological confirmation has been considered the gold standard for diagnosing the condition [6]. However, in recent years, noninvasive diagnostic approaches have become increasingly important. Symptomatic improvement during empirical treatment with oral contraceptives, when considered alongside imaging findings, may also further support a presumptive diagnosis of endometriosis [3]. A combination of clinical examination and imaging techniques is commonly used to improve diagnostic accuracy and facilitate preoperative surgical planning [7]. Digital rectal examination (DRE) may provide important information about posterior compartment involvement, particularly in patients with deeply infiltrating endometriosis [8].

Among imaging modalities, transvaginal ultrasonography (TVUS) is widely used as a first-line diagnostic tool due to its wide availability and high level of diagnostic accuracy for ovarian endometriomas and deeply infiltrating lesions [9]. Magnetic resonance imaging (MRI) may be used as an additional diagnostic modality, particularly when extensive disease is suspected or when detailed preoperative mapping of pelvic structures is required [10].

A structured and clinically meaningful classification of endometriosis is essential in order to accurately define the anatomical extent of the disease and organ involvement, particularly when there is deeply infiltrating endometriosis [11]. Standardized staging systems provide a common terminology that facilitates interdisciplinary communication among gynecologic surgeons, radiologists, colorectal specialists, and reproductive medicine experts [12, 13]. The #ENZIAN classification was introduced in 2021 as a comprehensive system allowing a detailed description of the anatomical compartments and extent of endometriosis, including deeply infiltrating lesions [13]. Originally developed for intraoperative staging, the classification has therefore been adapted for preoperative use (where it is termed “cENZIAN” or “c#ENZIAN” for clinical Enzian) [14]. Despite advances in preoperative diagnostic tools and classification systems, the clinical relevance of routinely using preoperative staging tools to predict surgical parameters remains unclear. In particular, it has not yet been sufficiently investigated whether the additional use of preoperative c#ENZIAN scoring and digital rectal examination improves the predictability of surgery or influences surgical outcomes in patients with endometriosis.

The aim of the present study was therefore to assess whether the preoperative use of c#ENZIAN scoring and digital rectal examination improved operative predictability and surgical outcomes in women undergoing surgery for endometriosis.

## Materials and methods

### Study population and study design

Women aged ≥ 18 years with suspected symptomatic endometriosis were prospectively included in the study. The patients were recruited consecutively between February 2024 and January 2026 at three tertiary gynecological centers in Germany: the Department of Obstetrics and Gynecology, Karlsruhe Municipal Hospital, Karlsruhe; the Department of Obstetrics and Gynecology, ViDia Hospital, Karlsruhe; and the Department of Obstetrics and Gynecology, Oberschwabenklinik, Ravensburg. Participants were enrolled and randomized before any study-related physical examination or diagnostic assessment was performed. The participants were randomly allocated to one of three study groups using a simple randomization procedure stratified by participating center. At each center, randomization was performed using the same standardized procedure. Allocation concealment was ensured by selecting one of three sealed, opaque and identical envelopes, each corresponding to a distinct study group. The envelopes were not accessible before participant inclusion and were opened only after eligibility had been confirmed and written informed consent had been obtained, thereby ensuring concealment until assignment. Participants were allocated to three groups as follows: Group 1 underwent DRE plus c#ENZIAN assessment, Group 2 underwent c#ENZIAN without DRE, and Group 3 underwent DRE without c#ENZIAN assessment. Data were collected in a pseudonymized manner using a standardized case report form. The variables recorded included patient age, body mass index (BMI), and the number of prior abdominal surgical procedures. The study protocol was approved by the Ethics Committee of the State Medical Association of Baden-Württemberg (ethics documentation number: F-2023-139). All participants provided written informed consent to the use of their clinical data for prospective data collection and analysis.

### Statistical analysis

The primary endpoint was the proportion of patients with an absolute difference of ≤ 10 min between the estimated and the actual operative time. All analyses were performed according to the intention-to-treat principle. Baseline characteristics were summarized using descriptive statistics. Continuous variables are presented as mean ± standard deviation, or median as appropriate, whereas categorical variables are presented as frequencies and percentages. The three study groups were compared using a mixed-effects logistic regression model, with study group included as a fixed effect and study center as a random effect to account for clustering by center. The results are reported as odds ratios (ORs) with corresponding 95% confidence intervals (CIs). Owing to the exploratory nature of this pilot study, the primary emphasis was placed on the estimation of effect sizes and 95% confidence intervals rather than formal hypothesis testing. Accordingly, *p* values were interpreted as exploratory. A two-sided p value < 0.05 was considered statistically significant. All statistical analyses were performed using JASP (version 0.95.4; University of Amsterdam and others).

### Sample size calculation

As this was designed as a prospective, randomized, multicenter pilot study, no formal sample size calculation was performed to demonstrate confirmatory efficacy. A total of 105 patients (35 per study group) were planned for inclusion. This sample size was considered sufficient to assess the feasibility of the study design, evaluate study procedures and recruitment, and provide preliminary estimates of event rates and effect sizes for the primary endpoint, thereby informing the sample size calculation of a subsequent adequately powered confirmatory multicenter trial.

### Primary end point

The largest amount of prior information was available for Group 1, and it was therefore hypothesized that in Group 1, the estimation of the operating time could be expected to be more precise than in Group 2 and Group 3. The primary endpoint was the proportion of operations with an absolute difference of ≤ 10 min between the estimated and the actual operative time. To minimize variability related to center-specific workflows and to ensure a standardized assessment of this outcome across all participating centers, operative time was defined as the time from skin incision to skin closure.

### Secondary end points

Categorical perioperative and postoperative outcomes (e.g., conversion rate, rate of abandonment of the operation, complete resection rate, planned secondary surgery, congruency between the preoperative Enzian assessment and intraoperative findings, postoperative changes in pain, and patient satisfaction) were compared using Chi-square or Fisher’s exact tests as appropriate.

## Results

### Baseline characteristics

Baseline characteristics are summarized descriptively for each study group in Table 1. Continuous variables are reported as means plus or minus standard deviation and categorical variables as counts and percentages. Baseline demographic and clinical characteristics were comparable across the three study groups. Mean age ranged from 31.6 to 35.1 years, and mean body mass index ranged from 24.1 to 24.4 kg/m^2^. The mean presurgery score was similar between groups. In line with the Consolidated Standards of Reporting Trials (CONSORT) recommendations for randomized-controlled trials, no hypothesis testing was conducted for baseline variables, since any between-group differences are expected to arise from random variation inherent to the randomization process.

**Table 1:** Baseline characteristics of the study population, showing mean and standard deviation (SD) for age, body mass index (BMI) and presurgery scores, and frequency and percentage (%) for other variables

Characteristic	Group 1 c#ENZIAN and DRE (*n* = 38)	Group 2 c#ENZIAN only (*n* = 34)	Group 3 DRE only (*n* = 35)
Age (years)	31.55 (6.9)	35.06 (8.0)	31.77 (7.0)
BMI (kg/m^2^)	24.11 (4.9)	24.19 (4.5)	24.45 (5.6)
Presurgery score (total for laparoscopy 1 point, laparotomy 2 points)	0.76 (1.01)	0.68 (0.83)	0.69 (1.02)
MRI (yes = 1, no = 0)	5 (13.2%)	6 (17.6%)	5 (14.3%)
Ultrasound (yes = 1, no = 0)	38 (100%)	34 (100%)	35 (100%)
DRE (yes = 1, no = 0)	38 (100%)	0 (0%)	35 (100%)
Suspected peritoneal endometriosis (P), *n* (%)	32 (84.21%)	27 (79.41%)	27 (77.14%)
Suspected ovarian endometriosis (O), *n *(%)	11 (28.95%)	13 (38.24%)	13 (37.14%)
Suspected DIE, *n* (%)	7 (18.42%)	7 (20.59%)	8 (22.85%)
Time interval between examination and surgery (days)	77.29 (46.28)	89.74 (45.13)	74.89 (30.79)

*BMI* body mass index; *DIE* deeply infiltrating endometriosis; *DRE* digital rectal examination; *MRI* magnetic resonance imaging

### Surgical parameters

**Fig. 1:**
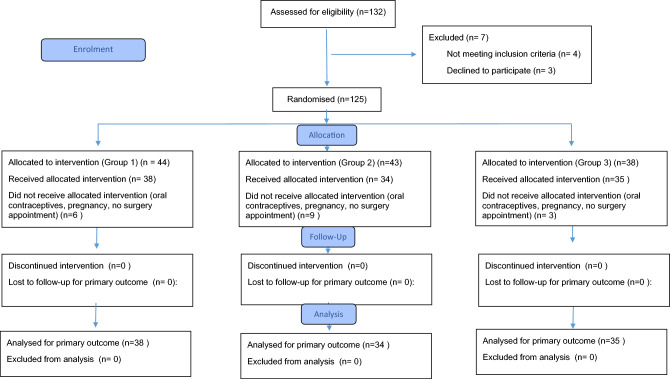
CONSORT 2025 flow diagram

A total of 107 patients were included in the analysis (Group 1: n = 38, Group 2: n = 34, Group 3: n = 35). Participant flow, including the numbers of randomized participants, those who received the allocated intervention and those included in the primary analysis, as well as losses and exclusions after randomization with corresponding reasons, is presented in Fig. 1. The primary endpoint was defined as an absolute difference of ≤ 10 min between the estimated and the actual operative time. The primary endpoint was achieved in 9 of 38 patients (23.7%) in Group 1, 16 of 34 patients (47.1%) in Group 2, and 15 of 35 patients (42.9%) in Group 3. Exploratory comparison of the three groups did not demonstrate a statistically significant difference in the proportion of accurate operative time estimations (χ^2^ = 4.85, p = 0.088) (Table 2). However, a numerical trend toward higher accuracy was observed in Groups 2 and 3 compared with Group 1. Given the pilot character of the study, these findings should be interpreted as estimates of effect size to inform the design rather than confirmatory evidence.

**Table 2:** Primary endpoint and accurate estimation of operative time (absolute error ≤ 10 min)

Group	Success ≤ 10 min	Percentage
1 (two informations)	9/38	23.7%
2 (one information)	16/34	47.1%
3 (one information)	15/35	42.9%

Exploratory comparison: Chi-square χ^2^ = 4.85, p = 0.088

The median absolute difference between estimated and actual operative time was 29.5 min in Group 1, 11.0 min in Group 2, and 14.0 min in Group 3. Mean absolute differences were 30.6, 28.8, and 23.6 min, respectively. Exploratory comparison using the Kruskal–Wallis test showed no statistically significant difference between the three study groups (p = 0.093), although a numerical trend toward smaller estimation errors was observed in Group 2 and 3 (Table 3).

**Table 3:** Absolute difference between estimated and actual operative time

Variable	Group 1 (*n* = 38)	Group 2 (*n* = 34)	Group 3 (*n* = 35)
Mean absolute error (min)	30.6	28.8	23.6
Median absolute error (min)	29.5	11.0	14.0
Standard deviation (min)	22.2	48.8	22.2

As the distribution was markedly skewed due to several extreme outliers (with absolute errors exceeding 200 min), the median was considered a more appropriate measure of central tendency than the mean

Exploratory comparison (Kruskal–Wallis test): p = 0.093

Multivariable logistic regression analysis adjusting for study center confirmed the exploratory findings. No statistically significant association between study group and accurate operative time estimation (absolute error ≤ 10 min) was observed. Compared with Group 1, Group 2 showed a trend toward a likelihood of achieving the primary endpoint, but the significance level was not reached, whereas no significant difference was found between Group 3 and 1 (results are shown in Table 4). In contrast, study center was independently associated with the primary endpoint. Compared with Center 1, patients treated at Centers 2 and 3 had significantly different odds of achieving an accurate operative time estimation.

**Table 4:** Multivariable logistic regression analysis

Variable	Odds Ratio	95%CI	*p*- value
Group 2 vs. Group 1	2.79	0.96–8.12	0.059
Group 3 vs. Group 1	1.76	0.60–5.16	0.304
Center 2 vs. Center 1	4.34	1.74–10.85	0.002
Center 3 vs. Center 1	4.90	1.13–21.23	0.034

*Endpoint*: accurate estimation of operative time (absolute error ≤ 10 min)

Conversion from laparoscopy to laparotomy did not occur in any patients in any of the study groups (0/107, 0%). No between-group comparisons were therefore performed. Intraoperative abandonment of the procedure occurred in one patient per group (Group 1: 1/38, 2.6%; Group 2: 1/34, 2.9%; Group 3: 1/35, 2.9%). Given the very small number of events, no formal statistical comparison was carried out. Secondary surgery was required in three patients overall (2.8%). None of the patients in Group 1 required repeat surgery (0/38, 0%), whereas two patients in Group 2 (2/34, 5.9%) and one patient in Group 3 (1/35, 2.9%) underwent secondary surgery. Given the low number of events, no formal statistical comparison was performed. Complete resection was achieved in 36 of the 38 patients (94.7%) in Group 1, 32 of the 34 patients (94.1%) in Group 2, and 34 of the 35 patients (97.1%) in Group 3. No statistically significant differences in complete resection rates were observed between the groups (χ^2^ test, *P* ≈ 0.87) (Table 5).

**Table 5:** Comparison of other different surgical findings between the groups

Characteristic	Group 1 c#ENZIAN and DRE (*n* = 38)	Group 2 c#ENZIAN only (*n* = 34)	Group 3 DRE only (*n* = 35)	*P* value
Conversion rate to laparotomy	0 (0%)	0 (0%)	0 (0%)	–
Rate of abandonment of surgery due to unsuspected findings	1 (2.6%)	1 (2.9%)	1 (2.9%)	–
Planned second operation	0 (0%)	2 (5.9%)	1 (2.9%)	–
Complete resection of endometriosis (R0)	36 (94.74%)	32 (94.11%)	34 (97.14%)	0.87

Data are shown as frequency and percentage (%). Multiple testing was not performed; therefore, Bonferroni correction was also not applied

*DRE* digital rectal examination

### Agreement of Enzian compartments

Agreement between the preoperative #ENZIAN assessments and intraoperative findings was compared between Group 1 (#ENZIAN score plus digital rectal examination) and Group 2 (#ENZIAN score only). The results are shown in Table 6.

**Table 6:** Comparison of estimated characteristics during the presurgical evaluation and surgical findings, using the prefix “c” for clinical findings and the prefix “s” for surgical findings, in accordance with the #ENZIAN classification system

Characteristic	Group 1 c#ENZIAN and DRE (*n* = 38)	Group 2 c#ENZIAN only (*n* = 34)	Group 3 DRE only (*n* = 35)	*P* value
Congruency between cP and sP	7 (18.42%)	14 (41.17%)	–	0.035
Congruency between cT and sT	34 (89.47%)	25 (73.53%)	–	0.08
Congruency between cO and sO	16 (42.11%)	17 (50%)	–	0.50
Congruency between cDIE and sDIE	27 (71.05%)	17 (50%)	–	0.066
Congruency between cF and sF	23 (60.52%)	20 (58.82%)	–	0.87

Differences between the Group 1 and 2 were assessed using Pearson’s chi-square test. When expected cell counts were below 5, Fisher’s exact test was applied. To control for multiple testing and limit inflation of the type I error rate, a Bonferroni correction was applied, resulting in an adjusted significance threshold of α = 0.01 (0.05/5)

*DIE* deeply infiltrating endometriosis; *F* further location (e.g.: A, adenomyosis uteri, B, bladder, I, intestine, U, ureter); *P* peritoneum; *T* fallopian tube; *O* ovary

### Postoperative pain perception

Improvement in postoperative pain was reported by 27 of 38 patients (71%) in Group 1, 24 of 34 patients (71%) in Group 2, and 23 of 35 patients (66%) in Group 3. Pain remained unchanged in 24%, 21%, and 23%, respectively, while worsening was reported by 8%, 9%, and 11% of patients. No statistically significant differences in patient-reported pain change were observed between the groups (χ^2^ test, P > 0.5) (Table 7).

**Table 7:** Postoperative changes in pain due to surgery and patient satisfaction, reported as frequency and percentage (%)

Characteristic	Group 1 c#ENZIAN and DRE (*n* = 38)	Group 2 c#ENZIAN only (*n* = 34)	Group 3 DRE only (*n* = 35)	*P* value
Change in postoperative pain due to surgery				
Better	27 (71%)	24 (71%)	23 (66%)	> 0.5
Indifferent	9 (24%)	7 (21%)	8 (23%)	
Worse	3 (8%)	3 (9%)	4 (11%)	
Satisfaction with treatment				
Satisfied	30 (78%)	25 (74%)	24 (69%)	> 0.2
Indifferent	4 (10.5%)	8 (23.5%)	6 (17.1%)	
Not satisfied	4 (10.5%)	1 (2.9%)	5 (14.3%)	

*DRE* digital rectal examination

### Patient satisfaction

Postoperative satisfaction was reported by 30 of the 38 patients (78.9%) in Group 1, 25 of the 34 patients (73.5%) in Group 2, and 24 of the 35 patients (68.6%) in Group 3. Indifferent responses were observed in 10.5%, 23.5%, and 17.1%, respectively, while dissatisfaction was reported by 10.5%, 2.9%, and 14.3% of the patients. No statistically significant differences in patient satisfaction were observed between the groups (χ^2^ test, *P* > 0.2) (Table 7).

## Discussion

This multicenter pilot study evaluated the impact of preoperative #ENZIAN scoring and digital rectal examination on operative predictability and surgical outcomes in women undergoing surgery for suspected endometriosis. Specifically, it was assessed whether enhanced preoperative anatomical characterization with the largest amount of prior information for Group 1 improved the prediction of operative time, agreement with the intraoperative findings, and clinical outcomes.

The results demonstrate that neither diagnostic strategy provided a measurable advantage in predicting operative time. Furthermore, no significant differences were observed between the study groups with regard to surgical outcomes, including complete resection rates, postoperative pain perception, and patient satisfaction. In addition, the extent of agreement between the preoperative #ENZIAN assessment and intraoperative findings did not differ significantly between the diagnostic strategies after adjustment for multiple comparisons. Interestingly, patients treated in Group 2 showed a higher likelihood of achieving the primary endpoint, and patients treated in center 2 and 3 also had significantly higher odds of achieving an accurate operative time estimation, so that center-specific factors may have had a greater influence on estimation accuracy than the intervention itself.

These results suggest that more detailed clinical classification and preoperative assessment do not necessarily translate into improved surgical decision-making and surgical outcome in endometrioses patients and other factors like center specific may be more relevant. This observation is consistent with the current evidence, which has identified transvaginal ultrasonography and magnetic resonance imaging as the cornerstones of noninvasive diagnosis and preoperative evaluation in patients with endometriosis [15, 16]. While classification systems such as the #ENZIAN score facilitate standardized reporting, their contribution to surgical planning remains uncertain [17–19]. Previous research has shown that endometriosis classification systems are primarily designed for anatomical description and demonstrate limited correlation with clinical outcomes—restricting their usefulness for guiding surgical planning [20]. Although imaging allows accurate assessment of the extent and localization of disease, this level of diagnostic precision does not necessarily translate into improved operative predictability or better patient outcomes.

In the present study, preoperative #ENZIAN scoring together with DRE did not improve the prediction of operative time, agreement with intraoperative findings, or patient-reported outcomes. Thus, increasing the complexity of preoperative classification and assessment does not appear to enhance surgical planning in a meaningful way. In contrast, operative time prediction was least accurate in the group with the most comprehensive preoperative information.

Most available data on pelvic examinations focus on vaginal assessment, with sparse evidence supporting the specific additional role of DRE [21, 22]. In comparison with the high diagnostic performance of transvaginal ultrasound and MRI, clinical examination—including digital rectal assessment—shows lower diagnostic accuracy (23, 24).

The very low rates of conversion, intraoperative discontinuation of the procedure, and secondary surgery observed in this study are also notable. These low event rates indicate that surgery was generally feasible and safe in all groups, but they also limit the ability to detect small between-group differences. Nevertheless, the absence of any consistent trend toward better outcomes in the intervention groups argues against a clinically meaningful benefit of the preoperative strategies that were tested.

## Strengths and limitations

The strengths of this study include its prospective, randomized, multicenter design, standardized data collection, and the prospective application of the latest #ENZIAN criteria, reflecting real-world clinical practice in specialized centers. However, several limitations must be acknowledged. Operative time was used as a primary surrogate for predictability; however, this metric is susceptible to institutional and personal factors. Second, although short-term patient-reported outcomes were assessed, long-term data on recurrence and fertility were not included, and these might be influenced by the precision of preoperative staging. The distribution of estimated and actual operative time was markedly skewed due to several extreme outliers, because no distinction was made between patients undergoing surgery for superficial endometriosis and those undergoing surgery for deep infiltrating endometriosis. Based on the effect size assumption used for planning, a future confirmatory trial would require at least 81 patients per group (n = 243) to achieve 80% statistical power. Finally, since the study was designed to compare diagnostic strategies rather than imaging modalities, the findings should not be interpreted as diminishing the value of expert ultrasonography or MRI in preoperative assessment.

## Conclusion

Simplifying preoperative protocols by omitting redundant diagnostic steps may reduce clinical complexity and patient discomfort without compromising the quality of care or surgical safety. Complex preoperative assessment and classification did not significantly improve the accuracy of operative time prediction in this pilot trial. Although statistical significance was not achieved, the observed effect size was clinically relevant. Compared with Group 1 (with the most preoperative information available), the proportion of accurate operative time estimations was 23.4 percentage points higher in Group 2 and 19.2 percentage points higher in Group 3 (both, with only one preoperative information available), corresponding to a medium size effect (Cohen’s h = 0.49 and 0.40, respectively). This pilot study suggests that providing additional preoperative information alone may not improve the accuracy of operative time estimation. Instead, operative complexity and center-specific factors appear to play a more important role. These findings should guide the design of future prediction models and prospective studies.

## Data Availability

All data supporting the findings of this study are available within the paper and its Supplementary Information.

## References

[1] Giudice LC (2010) Clinical practice. Endometriosis N Engl J Med 362(25):2389–2398 20573927 10.1056/NEJMcp1000274 PMC3108065

[2] Burney RO, Giudice LC (2012) Pathogenesis and pathophysiology of endometriosis. Fertil Steril 98(3):511–519 22819144 10.1016/j.fertnstert.2012.06.029 PMC3836682

[3] Becker CM, Bokor A, Heikinheimo O, Horne A, Jansen F, Kiesel L et al (2022) ESHRE guideline: endometriosis. Hum Reprod Open. 2022(2):hoac009 35350465 10.1093/hropen/hoac009 PMC8951218

[4] Bazot M, Darai E (2017) Diagnosis of deep endometriosis: clinical examination, ultrasonography, magnetic resonance imaging, and other techniques. Fertil Steril 108(6):886–894 29202963 10.1016/j.fertnstert.2017.10.026

[5] Nisenblat V, Prentice L, Bossuyt PM, Farquhar C, Hull ML, Johnson N (2016) Combination of the non-invasive tests for the diagnosis of endometriosis. Cochrane Database Syst Rev 7(7):CD012281 27405583 10.1002/14651858.CD012281 PMC6953325

[6] Tavcar J, Loring M, Movilla PR, Clark NV (2020) Diagnosing endometriosis before laparoscopy: radiologic tools to evaluate the disease. Curr Opin Obstet Gynecol 32(4):292–297 32398583 10.1097/GCO.0000000000000638

[7] Chapron C, Marcellin L, Borghese B, Santulli P (2019) Rethinking mechanisms, diagnosis and management of endometriosis. Nat Rev Endocrinol 15(11):666–682 31488888 10.1038/s41574-019-0245-z

[8] Moro F, Ianieri MM, De Cicco Nardone A, Carfagna P, Mascilini F, Vizzielli G et al (2024) Comparison of clinical and ultrasound examinations in assessing the parametria in patients with deep infiltrating endometriosis: a multicentre prospective study. Reprod Biomed Online 48(4):103733 38401251 10.1016/j.rbmo.2023.103733

[9] Goncalves MO, Siufi Neto J, Andres MP, Siufi D, de Mattos LA, Abrao MS (2021) Systematic evaluation of endometriosis by transvaginal ultrasound can accurately replace diagnostic laparoscopy, mainly for deep and ovarian endometriosis. Hum Reprod 36(6):1492–1500 33864088 10.1093/humrep/deab085

[10] Thomassin-Naggara I, Lamrabet S, Crestani A, Bekhouche A, Wahab CA, Kermarrec E et al (2020) Magnetic resonance imaging classification of deep pelvic endometriosis: description and impact on surgical management. Hum Reprod 35(7):1589–1600 32619220 10.1093/humrep/deaa103

[11] Lee SY, Koo YJ, Lee DH (2021) Classification of endometriosis. Yeungnam Univ J Med 38(1):10–18 32764213 10.12701/yujm.2020.00444 PMC7787892

[12] Johnson NP, Hummelshoj L, Adamson GD, Keckstein J, Taylor HS, Abrao MS et al (2017) World endometriosis society consensus on the classification of endometriosis. Hum Reprod 32(2):315–324 27920089 10.1093/humrep/dew293

[13] Keckstein J, Saridogan E, Ulrich UA, Sillem M, Oppelt P, Schweppe KW et al (2021) The #Enzian classification: a comprehensive non-invasive and surgical description system for endometriosis. Acta Obstet Gynecol Scand 100(7):1165–1175 33483970 10.1111/aogs.14099

[14] Enzelsberger SH, Oppelt P, Nirgianakis K, Seeber B, Drahonovsky J, Wanderer L et al (2022) Preoperative application of the Enzian classification for endometriosis (The cEnzian Study): a prospective international multicenter study. BJOG 129(12):2052–2061 35596694 10.1111/1471-0528.17235 PMC9796328

[15] Guerriero S, Saba L, Pascual MA, Ajossa S, Rodriguez I, Mais V et al (2018) Transvaginal ultrasound vs magnetic resonance imaging for diagnosing deep infiltrating endometriosis: systematic review and meta-analysis. Ultrasound Obstet Gynecol 51(5):586–595 29154402 10.1002/uog.18961

[16] Aas-Eng MK, Keckstein J, Condous G, Abrao MS, Hudelist G (2022) Deep endometriosis: can surgical complexity and associated risk factors be evaluated with transvaginal sonography and classification systems. Eur J Obstet Gynecol Reprod Biol 276:204–206 35930816 10.1016/j.ejogrb.2022.07.011

[17] Hudelist G, Montanari E, Salama M, Dauser B, Nemeth Z, Keckstein J (2021) Comparison between sonography-based and surgical extent of deep endometriosis using the Enzian classification - a prospective diagnostic accuracy study. J Minim Invasive Gynecol 28(9):1643–1649 33582378 10.1016/j.jmig.2021.02.009

[18] Montanari E, Bokor A, Szabo G, Kondo W, Trippia CH, Malzoni M et al (2022) Accuracy of sonography for non-invasive detection of ovarian and deep endometriosis using #Enzian classification: prospective multicenter diagnostic accuracy study. Ultrasound Obstet Gynecol 59(3):385–391 34919760 10.1002/uog.24833

[19] Condous G, Gerges B, Thomassin-Naggara I, Becker CM, Tomassetti C, Krentel H et al (2024) Non-invasive imaging techniques for diagnosis of pelvic deep endometriosis and endometriosis classification systems: an international consensus statement. J Minim Invasive Gynecol 31(7):557–573 38819341 10.1016/j.jmig.2024.04.006

[20] Vermeulen N, Abrao MS, Einarsson JI, Horne AW, Johnson NP, Lee TTM et al (2021) Endometriosis classification, staging and reporting systems: a review on the road to a universally accepted endometriosis classification. J Minim Invasive Gynecol 28(11):1822–1848 34690085 10.1016/j.jmig.2021.07.023

[21] Dabi Y, Fauconnier A, Rousset-Jablonski C, Tavenet A, Pizzofferrato AC, Deffieux X (2024) Do women with suspected endometriosis benefit from pelvic examination to improve diagnostic and management strategy? J Gynecol Obstet Hum Reprod 53(2):102724 38224817 10.1016/j.jogoh.2024.102724

[22] Chapron C, Dubuisson JB, Pansini V, Vieira M, Fauconnier A, Barakat H et al (2002) Routine clinical examination is not sufficient for diagnosing and locating deeply infiltrating endometriosis. J Am Assoc Gynecol Laparosc 9(2):115–119 11960033 10.1016/s1074-3804(05)60117-x

[23] Zhang X, He T, Shen W (2020) Comparison of physical examination, ultrasound techniques and magnetic resonance imaging for the diagnosis of deep infiltrating endometriosis: a systematic review and meta-analysis of diagnostic accuracy studies. Exp Ther Med 20(4):3208–3220 32855690 10.3892/etm.2020.9043 PMC7444323

[24] Nawrocka-Rutkowska J, Szydłowska I, Rył A, Ciećwież S, Ptak M, Starczewski A (2021) Evaluation of the diagnostic accuracy of the interview and physical examination in the diagnosis of endometriosis as the cause of chronic pelvic pain. Int J Environ Res Public Health. 10.3390/ijerph18126606 34205332 10.3390/ijerph18126606 PMC8296507

